# *N*-Hydroxyphthalimide exhibits antitumor activity by suppressing mTOR signaling pathway in BT-20 and LoVo cells

**DOI:** 10.1186/s13046-016-0315-1

**Published:** 2016-03-03

**Authors:** Min Wang, Ankun Zhou, Tao An, Lingmei Kong, Chunlei Yu, Jianmei Liu, Chengfeng Xia, Hongyu Zhou, Yan Li

**Affiliations:** State Key Laboratory of Phytochemistry and Plant Resources in West China, Kunming Institute of Botany, Chinese Academy of Sciences, Kunming, 650201 China; University of Chinese Academy of Sciences, Beijing, 100049 China

**Keywords:** *N*-Hydroxyphthalimide, mTOR, Inhibitor, Cancer, Mitochondrial apoptosis

## Abstract

**Background:**

*N*-Hydroxyphthalimide (NHPI), an important chemical raw material, was found to have potent and selective anti-proliferative effect on human breast carcinoma BT-20 cells, human colon adenocarcinoma LoVo and HT-29 cells during our screening for anticancer compounds. The purpose of this study is to assess the antitumor efficacy of NHPI *in vitro* and *in vivo* and to explore the underlying antitumor mechanism.

**Methods:**

Cell cytotoxicity of NHPI was evaluated using MTS assay and cell morphological analysis. After NHPI treatment, cell cycle, apoptosis and mitochondrial membrane potential were analyzed using flow cytometer. The subcellular localization of eukaryotic initiation factor 4E (eIF4E) was analyzed by immunofluorescence assay. The antitumor efficacy of NHPI *in vivo* was tested in BT-20 xenografts. The underlying antitumor mechanisms of NHPI *in vitro* and *in vivo* were investigated with western blot analysis in NHPI-treated cancer cells and tumor tissues. Statistical significance was determined using Student’s *t*-test.

**Results:**

*In vitro*, NHPI selectively inhibited the proliferation and induced G_2_/M phase arrest in BT-20 and LoVo cells, which was attributed to the inhibition of cyclin B1 and cdc2 expressions. Furthermore, NHPI induced apoptosis via mitochondrial pathway. Of note, NHPI effectively inhibited mammalian target of rapamycin (mTOR) complex 1 (mTORC1) and mTOR complex 2 (mTORC2) signaling, and overcame the feedback activation of Akt and extracellular signal-regulated kinase (ERK) caused by mTORC1 inhibition in BT-20 and LoVo cells. *In vivo*, NHPI inhibited tumor growth and suppressed mTORC1 and mTORC2 signaling in BT-20 xenografts with no obvious toxicity.

**Conclusions:**

We found for the first time that NHPI displayed antitumor activity which is associated with the inhibition of mTOR signaling pathway. Our findings suggest that NHPI may be developed as a promising candidate for cancer therapeutics by targeting mTOR signaling pathway and as such warrants further exploration.

## Background

Mammalian target of rapamycin (mTOR) is an atypical serine/threonine kinase that belongs to the phosphoinositide 3-kinase (PI3K)-related kinase family [1]. The mTOR kinase exists and acts as the catalytic subunit in two functionally and structurally distinct multi-protein complexes: mTOR complex 1 (mTORC1) and mTOR complex 2 (mTORC2) [2, 3]. The mTOR signaling pathway integrates both intracellular and extracellular signals and serves as a central regulator of cell metabolism, growth, proliferation and survival [1, 4]. The activation of mTORC1 leads to phosphorylation of ribosomal S6 protein kinase 1 (S6K1) and translational repressor eukaryotic initiation factor 4E (eIF4E)-binding protein 1 (4E-BP1), thus modulating ribosome biogenesis and the translation of proteins that promote cell growth [5]. S6K1 phosphorylates ribosomal protein S6 (S6) and enhances the translation of mRNAs [1, 6]. S6K1 also phosphorylates other important targets, including insulin receptor substrate 1 (IRS-1), eukaryotic initiation factor 4B, programmed cell death 4, eukaryotic elongation factor-2 kinase and mTOR [6]. Phosphorylation of 4E-BP1 releases its inhibitory effect on eIF4E, promoting cap-dependent mRNA translation [7]. eIF4E enhances cell growth, proliferation, survival and angiogenesis by selectively translating mRNA such as cyclin D1, Bcl-2, Bcl-xL and vascular endothelial growth factor [8, 9]. mTORC2 regulates cell survival by phosphorylating on Ser473 of Akt, also known as protein kinase B, which is one of the most important survival kinases [1, 10].

Consistent with a critical role in regulating cell growth and metabolism, dysregulation of the mTOR signaling is commonly observed in human cancers [11–14]. Aberrant mTOR signaling pathway activation through oncogene stimulation or loss of tumor suppressors contributes significantly to cancer initiation, development and chemotherapy resistance [1, 7, 11, 14–18]. Cumulative evidence indicates that mTOR signaling pathway has become an attractive target for cancer therapy and targeting mTOR signaling has been exploited as a promising tumor-selective therapeutic strategy [11, 12, 19, 20]. The most well-characterized inhibitors targeting this pathway are rapamycin and its analogs (also referred as rapalogs), which are currently used with success for treating certain types of tumors [21, 22]. However, rapalogs incompletely inhibit mTORC1 and generally fail to inhibit mTORC2 in some tumor types, which may not be sufficient for achieving a robust anticancer effect [15, 21, 23, 24]. Moreover, resistance to treatment with rapalogs has been reported. The resistance may be associated with disrupting the mTORC1-mediated negative feedback loops to IRS-1/PI3K, mTORC2 and mitogen-activated protein kinase (MAPK)/extracellular signal-regulated kinase (ERK), which lead to activation of Akt and MAPK/ERK signaling, thereby, counteracting the antitumor potential of mTORC1 inhibition [24–27]. These insights have prompted the development of potent mTOR signaling inhibitors capable of overcoming the feedback activation of several oncogenic pathways and fully inhibiting the function of both mTOR complexes.

In the present study, *N*-Hydroxyphthalimide (NHPI), an important chemical raw material, was found to have potent and selective anti-proliferative effect on human breast carcinoma BT-20 cells, human colon adenocarcinoma LoVo and HT-29 cells during our screening for anticancer compounds. *In vitro*, NHPI induced G_2_/M phase arrest in BT-20 and LoVo cells, which was attributed to the inhibition of cyclin B1 and cdc2 expressions. Moreover, NHPI induced apoptosis via mitochondrial pathway. Of note, NHPI effectively inhibited mTORC1 and mTORC2 signaling in BT-20 and LoVo cells, negating the feedback activation of Akt and ERK caused by mTORC1 inhibition. *In vivo*, NHPI significantly inhibited tumor growth and suppressed mTORC1 and mTORC2 signaling in BT-20 xenografts with no obvious toxicity. These findings suggest that NHPI may be a potential candidate for cancer therapeutics by targeting mTOR signaling pathway and as such warrants further exploration.

## Methods

### Reagents

*N*-Hydroxyphthalimide was purchased from Accela ChemBio Co., Ltd. (Shanghai, China). Propidium iodide (PI), RNase A and 4,6-diamidino-2-phenylindole (DAPI) were from Sigma-Aldrich. Antibodies of mTOR, Phospho-mTOR (Ser2448), Phospho-mTOR (Ser2481), S6K1, Phospho-S6K1 (Thr389), Phospho-S6 Ribosomal Protein (Ser235/236), 4E-BP1, Phospho-4E-BP1 (Ser65), Phospho-Akt (Ser473), Phospho-Akt (Thr308), Cleaved PARP, Cleaved Caspase 3, Caspase 9, cyclin B1, cdc2 were obtained from Cell Signaling Technology; antibodies of S6, Akt, P-ERK1/2, β-actin, Caspase 3, Caspase 8, Bcl-xL, survivin were from Santa Cruz; antibody of ERK was from Epitomics; antibody of eIF4E was obtained from BD Biosciences; Alexa Fluor® 647 donkey anti-mouse IgG antibody was purchased from Invitrogen; all the other secondary antibodies were from Sigma-Aldrich.

### Cell culture

Human breast carcinoma cell line BT-20 and human normal breast epithelial cell line MCF-10A were purchased from Cell Research Center, IBMS, CAMS/PUMC (Beijing, China). Human breast cancer cell lines SK-BR-3, MDA-MB-231, MDA-MB-468, MDA-MB-436, HCC1937 and MCF7, human colon cancer cell lines LoVo, HT-29, Caco-2 and RKO, human leukemia cell lines Jurkat, HL-60 and K-562, human ovary adenocarcinoma cell line NIH:OVCAR-3 and human hepatocellular carcinoma cell line SMMC-7721 were purchased from Cell Bank of Type Culture Collection of Chinese Academy of Sciences (Shanghai, China). All cells were cultured according to the vendor’s instructions.

### Cell viability assay

Cell viability was determined by MTS assay, according to the protocol of CellTiter 96®AQ_ueous_ One Solution Cell Proliferation Assay kit (Promega). Briefly, 100 μl of cell suspensions were seeded into each well of 96-well plates at a density of 5 × 10^3^ cells/well and cultured overnight. Cells were then exposed to the tested compounds in triplicates for 48 h, 20 μL of CellTiter 96®AQ_ueous_ One Solution Reagent was added to each well, and the cells were further incubated at 37 °C for 1–2 h. Cell viability was detected by measuring the optical density (OD) at 490 nm using a microplate reader (Bio-Rad Laboratories). The half inhibitory concentration (IC_50_) was determined by the relative survival curve.

### Cell morphological analysis

Cells were seeded in 6-well plates (4 × 10^5^ cells/well) and grown overnight. Next day, the cells were treated with indicated concentrations of NHPI. After incubation for 24 h, the morphology of cells was observed using a phase-contrast microscope (Eclipse Ti, Nikon) at 100× magnification.

### Cell cycle analysis

Cells were seeded in 6-well plates and incubated at 37 °C overnight. Cells were then treated with indicated concentrations of NHPI for 24 h. Subsequently, the cells were collected and fixed with pre-cold 70 % ethanol overnight at −20 °C. Fixed cells were washed with PBS and incubated with a staining solution containing RNase A and PI in PBS for 30 min at room temperature. Fluorescence intensity was analyzed by FACSCalibur flow cytometer (BD Biosciences). The distribution of cells in each phase of the cell cycle was determined using FlowJo7.6.1 analysis software.

### Apoptosis assay

Cell apoptosis was analyzed using the Annexin V-FITC Apoptosis Detection Kit I (BD Biosciences) according to the manufacturer’s protocol. Cells were seeded in 6-well plates at a density of 1 × 10^5^ cells/well and incubated overnight. After indicated treatment, cells were collected and washed twice with cold PBS, then resuspended in a binding buffer containing Annexin V-FITC and PI. After incubation for 15 min at room temperature in the dark, the fluorescence intensity was measured using a FACSCalibur flow cytometer (BD Biosciences).

### Protein extraction and western blot analysis

Cells were seeded in 6-well plates at a density of 4 × 10^5^ cells/well. The next day, cells were treated with indicated concentrations of NHPI for 24 h. Following treatment, cells were washed with cold PBS. On ice, cells were lysed in RIPA buffer, containing 50 mM Tris, pH 7.4; 150 mM NaCl; 1 % sodium deoxycholate; 0.1 % sodium dodecyl sulfate; 1 % NP-40; 1 mM EDTA; 1 mM PMSF; protease and phosphatase inhibitor cocktail (Roche). Lysates were centrifuged, and the supernatant was dissolved with 5× sample loading buffer and boiled for 5 min. Protein extracts were quantitated and loaded on 8–12 % sodium dodecyl sulfate polyacrylamide gel, electrophoresed and transferred to polyvinylidene fluoride membranes (Millipore). Membranes were incubated with 5 % non-fat dry milk to block non-specific binding and were incubated with primary antibodies, then with appropriate secondary antibodies conjugated to horseradish peroxidase. Proteins of interest were incubated with Pierce ECL substrate (Thermo Scientific) and visualized by chemiluminescent detection on an ImageQuant LAS 4000 mini (GE Healthcare).

### Mitochondrial membrane potential assay

The mitochondria membrane potential (MMP) in BT-20 cells was measured using JC-1 Mitochondrial Membrane Potential Assay Kit (Cayman Chemical Company). Briefly, BT-20 cells were seeded in 6-well plates at a density of 2 × 10^5^ cells/well and incubated overnight for cell attachment. Cells were then treated with indicated concentrations of NHPI. After 48 h, added 200 μl of the JC-1 Staining Solution (1:10 dilution in culture medium) into each well and incubated for 30 min at 37 °C in a CO_2_ incubator. Next, the cells were trypsinized and collected. The samples were analyzed immediately using a FACSCalibur flow cytometer (BD Biosciences).

### Immunofluorescence assay

BT-20 cells were seeded and cultured in 96-well plates (1 × 10^4^ cells/well) overnight and then treated with indicated concentrations of NHPI. After incubation for 6 h, cells were fixed with 4 % paraformaldehyde for 20 min, and the fixed cells were permeabilized with 0.1 % Triton X-100 for 10 min. After blocking with 2 % BSA at 37 °C for 30 min, cells were incubated with eIF4E antibody (1:400 in 2 % BSA) at 4 °C overnight and then the cells were incubated with Alexa Fluor® 647 donkey anti-mouse IgG antibody (1:1000 in 2 % BSA) for 1 h at room temperature. DAPI was used to stain the nuclei. To monitor the subcellular location of eIF4E, Cellomics® ArrayScan® V^TI^ HCS Reader (Thermo Scientific) was used to capture the images.

### In vivo studies

Three-week old female BALB/c nude mice were purchased from Vital River Laboratory Animal Technology (Beijing, China) and fed in a specific pathogen-free environment and treated in accordance with the guidelines of the Animal Ethics Committee of Kunming Institute of Botany (Kunming, China). 6.8 × 10^6^ BT-20 cells in Matrigel (Corning) were subcutaneously implanted into the right flank of nude mice for tumor formation. Tumor size was measured every three days in two dimensions with a digital caliper and calculated using the formula (length × width × width × 0.5). When the tumor reached approximately 100 mm^3^, mice were randomly divided into the control and treatment groups (*n* = 9/group). Subsequently, mice were treated daily by intraperitoneal injection with NHPI (40 mg/kg) prepared in a solution (10 % ethanol and 30 % PEG 400 in sterile saline) or vehicle control. At the end of treatment, mice were sacrificed and tumors were removed from mice. Then the tumors were weighed and frozen immediately at −80 °C for subsequent western blot analysis. Tumor samples were minced and homogenized to extract whole cell lysates. The clarified supernatants of the samples were applied for western blot analysis using specific antibodies.

### Statistical analysis

Results in *in vivo* studies were expressed as mean ± standard error (mean ± SE) and all the other results were expressed as mean ± standard deviation (mean ± SD). The data were analyzed using Student’s *t*-test. A probability value of *P* < 0.05 was considered to be statistically significant.

## Results

### Cytotoxicity of NHPI toward tumor cells

**Fig. 1:**
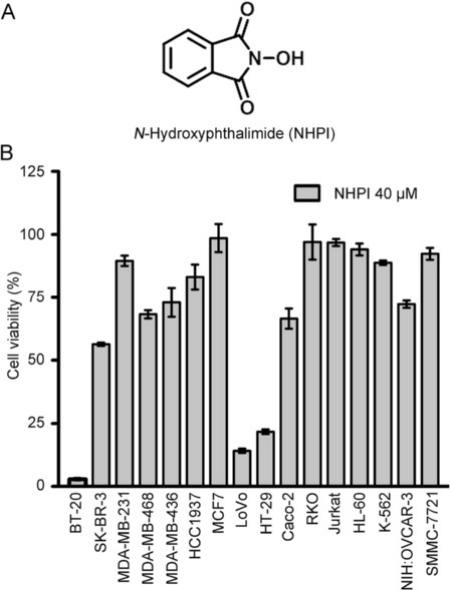
NHPI selectively decreases cell viability in BT-20, LoVo and HT-29 cells. **a** Chemical structure of NHPI. **b** All the tested cancer cells were treated with 40 μM NHPI for 48 h. Cell viability was determined by MTS assay and represented with relative viability versus control. Results were presented as mean ± SD (*n* = 3)

**Fig. 2:**
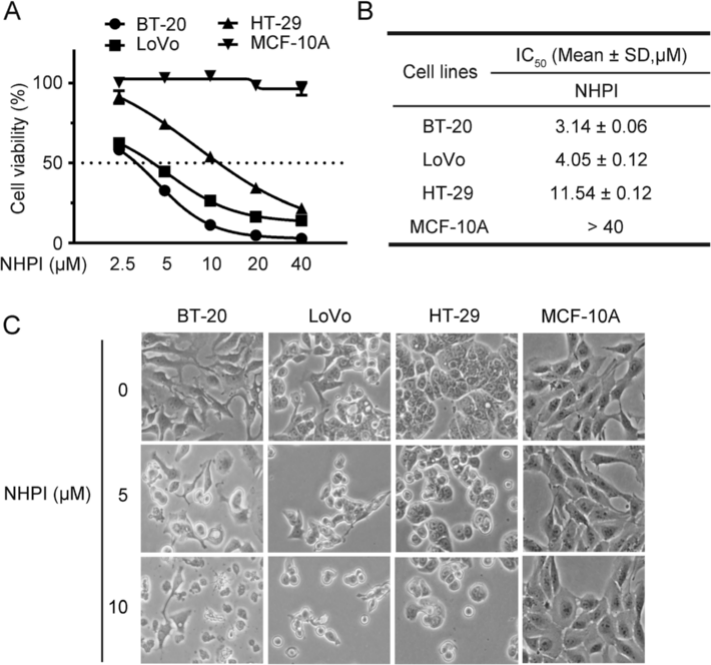
NHPI concentration-dependently decreases cell viability in BT-20, LoVo and HT-29 cells. **a** BT-20, LoVo, HT-29 and MCF-10A cells were treated with indicated concentrations of NHPI for 48 h. Cell viability was determined by MTS assay and represented with relative viability versus control. Results were presented as mean ± SD (*n* = 3). **b** IC_50_ values of NHPI on cell proliferation were shown with mean ± SD (*n* = 3). **c** The morphology of BT-20, LoVo, HT-29 and MCF-10A cells treated with NHPI at 5 and 10 μM for 24 h were observed under a phase-contrast microscope and photographed (100×)

During the screening for anticancer compounds, NHPI (Fig. 1a) was found to have potent and selective cytotoxicity against human breast carcinoma BT-20 cells, human colon adenocarcinoma LoVo and HT-29 cells (Fig. 1b). The anti-proliferative effect of NHPI on these three cancer cell lines and human normal breast epithelial MCF-10A cells was further tested using MTS assay by treating the cells with different concentrations of NHPI. As shown in Fig. 2a, treatment with NHPI (0–40 μM) for 48 h inhibited the proliferation of BT-20, LoVo and HT-29 cells in a concentration-dependent manner. IC_50_ value of NHPI on the proliferation of these three cancer cell lines was 3.14 ± 0.06 μM, 4.05 ± 0.12 μM and 11.54 ± 0.12 μM, respectively (Fig. 2b). Of note, NHPI did not affect the proliferation of human normal breast epithelial MCF-10A cells. Morphological changes were then observed in these cell lines after incubation with indicated concentrations of NHPI for 24 h. As illustrated in Fig. 2c, BT-20, LoVo and HT-29 cells exposed to NHPI exhibited distinct cell shrinkage and rounded shapes in a concentration-dependent manner. However, NHPI did not affect the morphology of human normal breast epithelial MCF-10A cells. Taken together, NHPI selectively inhibited the proliferation of BT-20, LoVo and HT-29 cells, suggesting that NHPI could be a promising anticancer agent for human breast carcinoma and human colon adenocarcinoma selective treatment.

### NHPI induces G_2_/M phase cell cycle arrest by inhibiting the expressions of cyclin B1 and cdc2

**Fig. 3:**
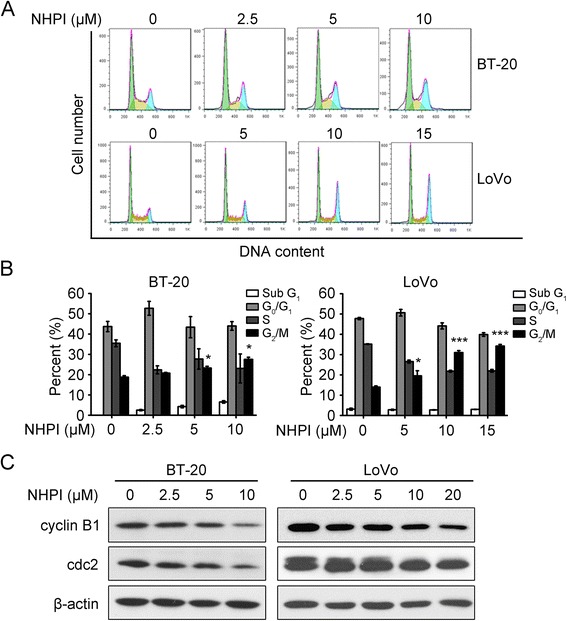
NHPI induces G_2_/M phase cell cycle arrest by inhibiting the expressions of cyclin B1 and cdc2. **a** BT-20 cells were treated with NHPI at 2.5, 5 and 10 μM for 24 h. LoVo cells were incubated with NHPI at 5, 10 and 15 μM for 24 h. Cells were stained with PI and subjected to flow cytometry analysis. Representative images were shown. **b** Quantitative analysis of cells in each cell cycle phase was performed. Results were presented as mean ± SD (*n* = 3). *p < 0.05 and ***p <0.001, difference versus 0 μM control group. **c** The inhibition of cyclin B1 and cdc2 expressions contributed to NHPI-induced G_2_/M phase arrest. Cells were treated with indicated concentrations of NHPI for 24 h, and the expression levels of G_2_/M phase regulatory proteins were analyzed by western blot analysis. β-actin was used as a loading control

Cell proliferation is controlled by the progression of the cell cycle [28]. To understand how NHPI inhibited cell proliferation, we assessed the effect of NHPI on cell cycle distribution using PI staining and flow cytometry. As shown in Fig. 3a, both BT-20 and LoVo cells were arrested in G_2_/M phase after incubation with various concentrations of NHPI for 24 h, especially at the high concentration. The percent of cells in the G_2_/M fraction significantly increased compared with the control accompanied by a decrease of cell number in S phase of the cell cycle (Fig. 3b).

To further understand the mechanism for NHPI-induced G_2_/M phase cell cycle arrest, the expression levels of key regulators of cell cycle were examined. Cyclin B1 and cdc2 (CDK1) are two key regulators for G_2_ to M phase transition [29]. As shown in Fig. 3c, treatment of BT-20 and LoVo cells with NHPI for 24 h repressed cellular protein expressions of cyclin B1 and cdc2 in a concentration-dependent manner. Taken together, these results demonstrate that NHPI arrests BT-20 and LoVo cells in G_2_/M phase of cell cycle in a concentration-dependent manner, with the involvement of decreasing the expressions of cyclin B1 and cdc2.

### NHPI induces apoptosis via mitochondrial pathway

**Fig. 4:**
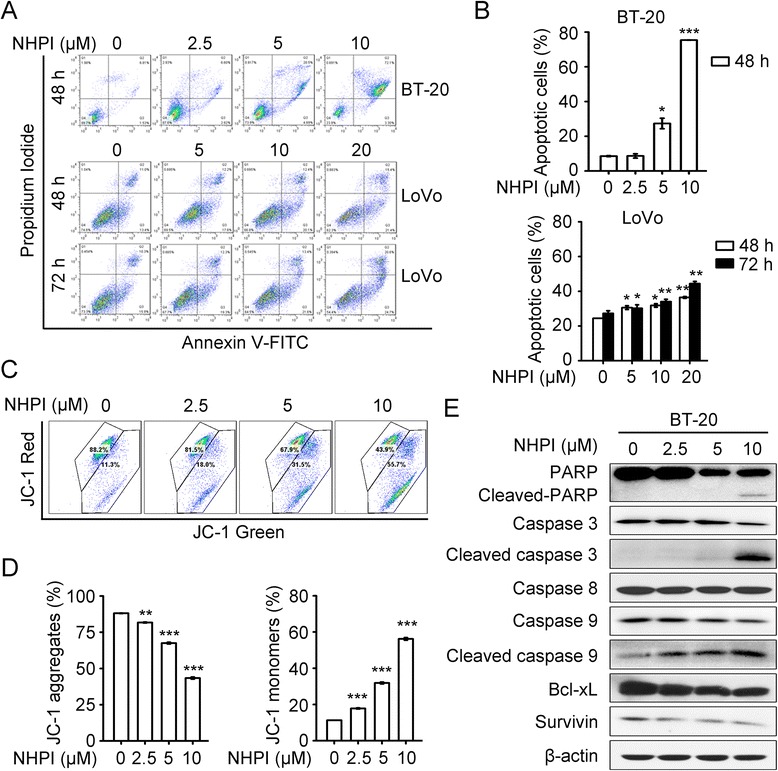
NHPI induces apoptosis via mitochondrial pathway. **a** BT-20 cells were treated with NHPI at 2.5, 5 and 10 μM for 48 h. LoVo cells were incubated with NHPI at 5, 10 and 20 μM for 48 h or 72 h. Cell apoptosis was analyzed by flow cytometry using the Annexin V-FITC and PI double staining. Representative images were presented. **b** Quantification of flow cytometry analysis of apoptosis. Results were presented as mean ± SD (*n* = 3). *p < 0.05, **p < 0.01 and ***p <0.001, difference versus 0 μM control group. **c** NHPI induced MMP loss in BT-20 cells. BT-20 cells were treated with NHPI at 2.5, 5 and 10 μM for 48 h, stained with JC-1 and subjected to flow cytometry analysis. The dot-plot representation of the flow cytometry analysis shows the distribution of JC-1 aggregates (cells emitting red fluorescence detected in the FL2 channel) and JC-1 monomers (cells emitting green fluorescence detected in the FL1 channel). **d** Histograms showing the percentage of JC-1 aggregate-positive and JC-1 monomer-positive cells. Results were presented as mean ± SD (*n* = 3). **p < 0.01 and ***p <0.001, difference versus 0 μM control group. **e** Effect of NHPI on the expressions of apoptosis-related proteins. Cells were treated with indicated concentrations of NHPI for 24 h, followed by western blot analysis with indicated antibodies. β-actin was used as a loading control

To determine whether the anti-proliferative effect of NHPI was associated with the induction of apoptosis, the Annexin V-FITC/PI double staining and flow cytometry analysis were used to analyze apoptosis parameter. The late and early apoptotic cells, which are shown, respectively, in the upper right and lower right quadrants of the dot plot, were counted as apoptotic cells. As shown in Fig. 4a, treatment of BT-20 cells with NHPI for 48 h increased the percentage of apoptotic cells in a concentration-dependent manner. When BT-20 cells were treated with NHPI at 10 μM, the total percentage of apoptotic cells increased about 8.8-fold compared with the vehicle control (Fig. 4b). Treatment of LoVo cells with NHPI increased the percentage of apoptotic cells in a concentration-and time-dependent manner (Fig. 4a and b).

Intrinsic apoptosis is also known as mitochondrial apoptosis because it depends on factors released from the mitochondria [30]. The mitochondrion-mediated pathway begins with the loss of mitochondrial membrane potential (MMP) [31, 32]. To determine whether MMP change was involved in NHPI-induced apoptosis, MMP change was measured by JC-1 staining. BT-20 cells were treated with NHPI for 48 h, stained with JC-1 and subjected to flow cytometry analysis. JC-1 forms aggregates, which emit red fluorescence in the mitochondria of healthy cells. However, it remains as monomers that exhibit green fluorescence during the loss of MMP. As shown in Fig. 4c and d, treatment of BT-20 cells with NHPI resulted in a significant increase of JC-1 monomers and a significant decrease of JC-1 aggregates, indicating that NHPI induced MMP loss in a concentration-dependent manner.

Apoptosis is also known as programmed cell death and caspases are the major executioners of apoptosis. Among the members of the caspase family, caspase-9 is the essential initiator required for mitochondrial apoptosis pathway [30, 33]. The mitochondrial pathway is regulated by the Bcl-2 family members which has both anti-apoptotic (Bcl-2 and Bcl-xL) and pro-apoptotic (Bax, Bad and Bid) proteins [34, 35]. To investigate the mechanism by which NHPI induced apoptosis, the expressions of apoptosis-related proteins were tested by western blot analysis. As shown in Fig. 4e, treatment of BT-20 cells with NHPI for 24 h induced a decline in anti-apoptotic proteins survivin and Bcl-xL as well as the activation of caspase 9 and caspase 3, but not caspase 8. In addition, NHPI increased the level of poly ADP-ribosepolymerase (PARP) cleavage, a known cellular substrate of caspases that plays vital role in apoptosis (Fig. 4e) [36]. Taken together, the above results suggest that NHPI induces apoptosis via mitochondrial pathway in BT-20 cells.

### NHPI inhibits both mTORC1 and mTORC2 signaling in BT-20 and LoVo cells

**Fig. 5:**
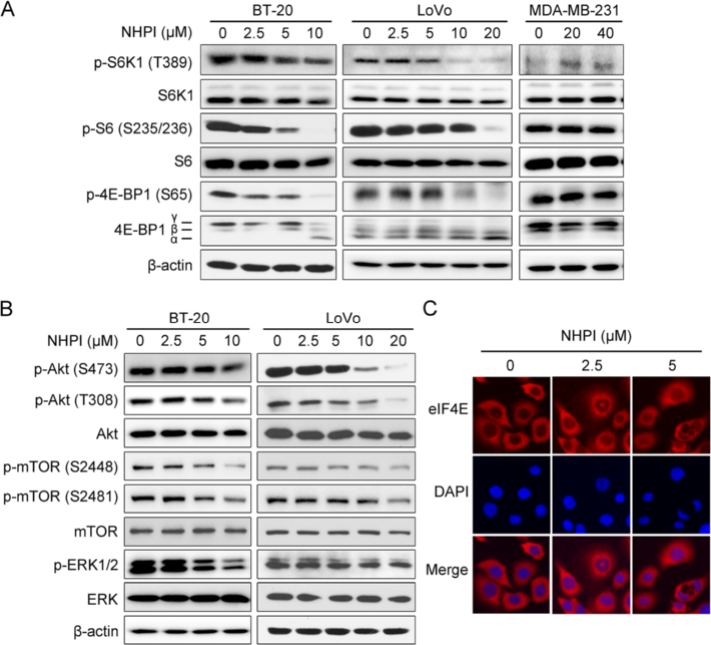
NHPI inhibits both mTORC1 and mTORC2 signaling in BT-20 and LoVo cells. **a** BT-20, LoVo and MDA-MB-231 cells were treated with indicated concentrations of NHPI for 24 h, followed by western blot analysis with indicated antibodies. β-actin was used as a loading control. **b** BT-20 and LoVo cells were treated with indicated concentrations of NHPI for 24 h, followed by western blot analysis with indicated antibodies. β-actin was used as a loading control. **c** NHPI induced the eIF4E nuclear translocation in BT-20 cells. BT-20 cells were incubated with indicated concentrations of NHPI for 6 h. Then the cells were analyzed by immunofluorescence assay labeling with eIF4E antibody and DAPI. eIF4E and nucleus were recognized by the red and blue fluorescence, respectively

The mTOR signaling pathway is pivotal in regulating major cell functions including cell growth, proliferation and survival [1, 4]. In our study, NHPI was found to selectively inhibit the proliferation and induced apoptosis in BT-20 and LoVo cells. We hypothesized that NHPI might be disrupting these cellular processes by primarily repressing the mTOR signaling pathway. To test this hypothesis, we set out to examine the effect of NHPI on mTOR signaling pathway in BT-20, LoVo and MDA-MB-231 cells. By western blot analysis, we found that NHPI inhibited phosphorylation of S6K1 and 4E-BP1, two best characterized direct substrates of mTORC1 [37], in a concentration-dependent manner in BT-20 and LoVo cells (Fig. 5a). Consistently, NHPI suppressed phosphorylation of S6, a substrate of S6K1 as well (Fig. 5a). Interestingly, NHPI at high concentration (40 μM) neither affected the cell viability nor inhibited mTORC1 signaling in MDA-MB-231 cells (Figs. 1b and 5a). The above data suggest that NHPI inhibits cell proliferation, at least in part, by disrupting the mTOR signaling pathway.

In addition, NHPI restrained phosphorylation of mTOR at Ser2448, a site phosphorylated by S6K1 [38], in a concentration-dependent manner in BT-20 and LoVo cells (Fig. 5b). Because Akt phosphorylation by PI3K is crucial for cell proliferation, growth and survival, we examined whether the PI3K site on Akt (Thr308) was also affected by NHPI. As shown in Fig. 5b, NHPI dramatically curbed PI3K-mediated Akt phosphorylation at Thr308. It is reported that the nuclear accumulation of eIF4E occurs in a 4E-BP-dependent manner specifically upon inhibition of mTOR signaling [39]. As shown in Fig. 5c, treatment of BT-20 cells with NHPI for 6 h induced the eIF4E nuclear translocation in a concentration-dependent manner. These results strongly prove that NHPI suppresses mTORC1 signaling.

mTOR functions as two complexes called mTORC1 and mTORC2. mTORC2 specifically senses growth factors and regulates proliferation, actin cytoskeleton, and cell survival [40, 41]. mTORC2 controls several members of the AGC subfamily of kinases including Akt, serum- and glucocorticoid-induce protein kinase 1 (SGK1), and protein kinase C-α (PKC-α) [1]. Akt regulates cellular processes such as survival, apoptosis, growth, and proliferation through the phosphorylation of several effectors [1, 11, 42]. Akt phosphorylates and inhibits tuberous sclerosis complex 2 (TSC2) to activate signaling through mTORC1 [11]. mTORC2 directly activates Akt by phosphorylating its hydrophobic motif (Ser473), a site required for its maximal activation [1, 10]. Akt is therefore both an upstream activator of mTORC1 and a downstream effector of mTORC2. We then examined whether NHPI had inhibitory effect on mTORC2 signaling. Indeed, NHPI repressed Akt phosphorylation at Ser473, the mTORC2 target site in BT-20 and LoVo cells (Fig. 5b). Phosphorylation of mTOR at Ser2481 is indicative of mTORC2 activation [43]. As shown in Fig. 5b, NHPI decreased phosphorylation of mTOR at Ser2481 as well.

Inhibition of mTORC1, such as by rapalogs, blocks the S6K-mediated negative feedback loops, which further promote activation of Akt and the MAPK/ERK signaling cascade, thus promoting cell proliferation, growth, and survival [15, 24, 25, 27]. Of note, NHPI suppressed phosphorylation of ERK1/2 in a concentration-dependent manner in BT-20 and LoVo cells (Fig. 5b). Taken together, these results demonstrate that NHPI inhibits both mTORC1 and mTORC2 signaling and overcomes feedback activation of Akt and ERK, indicating that NHPI may achieve a robust anticancer effect.

### NHPI inhibits tumor growth in human breast xenografts in association with suppression of both mTORC1 and mTORC2 signaling

**Fig. 6:**
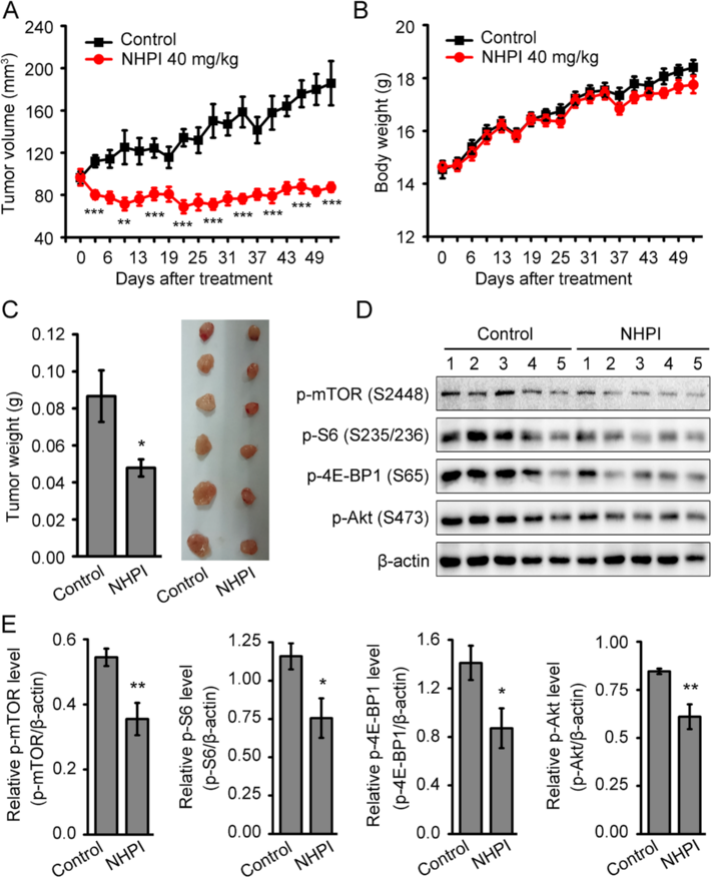
NHPI inhibits tumor growth in human breast xenografts in association with suppression of both mTORC1 and mTORC2 signaling. 6.8 × 10^6^ BT-20 cells in Matrigel were subcutaneously implanted into nude mice. When tumor size reached around 100 mm^3^, tumor-bearing mice were randomized into two groups and treated daily by intraperitoneal injection with 40 mg/kg NHPI or vehicle control for 53 days. **a** NHPI significantly inhibited tumor growth in BT-20 xenografts. The individual tumor volume was measured at indicated time (days) and presented as mean ± SE (*n* = 9). **p < 0.01 and ***p <0.001, difference versus vehicle-treated control group. **b** The individual body weight was measured at indicated time (days) and presented as mean ± SE (*n* = 9). **c** Tumors were removed from mice and tumor weight was measured at the end of treatment. Representative tumor images were shown and tumor weight was presented as mean ± SE (*n* = 9). *p < 0.05, difference versus vehicle-treated control group. **d** NHPI inhibits both mTORC1 and mTORC2 signaling in BT-20 tumor tissues. The phosphorylation levels of mTOR at Ser2448, S6 at Ser235/236, 4E-BP1 at Ser65 and Akt at Ser473 in tumor tissues were determined by western blot analysis. β-actin was used as a loading control. **e** Relative densitometric quantification of protein expression detected in (**d**). Results were presented as mean ± SE (*n* = 5). *p < 0.05 and **p < 0.01, difference versus vehicle-treated control group

The *in vitro* data presented above prompted us to further test the antitumor efficacy of NHPI *in vivo*. Tumor xenografts were established through subcutaneous inoculation with BT-20 cells in nude mice. When tumor size reached around 100 mm^3^, tumor-bearing mice were randomized into two groups and treated daily by intraperitoneal injection with 40 mg/kg NHPI or vehicle control for 53 days. NHPI treatment did not display any adverse effect on mice body weight in comparison with control group during the experimental period, indicating the safety of NHPI (Fig. 6b). However, treatment with 40 mg/kg NHPI resulted in a significant reduction in both tumor volume (Fig. 6a) and tumor weight (Fig. 6c) compared with the vehicle control. As shown in Fig. 6a, NHPI inhibited BT-20 xenografts growth starting from day 3 of treatment until the end of the study. At the end of treatment, the average tumor volume of vehicle control group was approximately 186 mm^3^ and that of NHPI-treated group was about 87 mm^3^, showing a 53 % tumor growth inhibition (Fig. 6a). Meanwhile, NHPI decreased 45 % of tumor weights in BT-20 xenografts, compared with vehicle control (Fig. 6c). These results demonstrate that NHPI has potent antitumor activity in BT-20 xenografts.

To find out whether mTOR signaling was also repressed by NHPI in BT-20 xenografts, tumor tissue extracts were subjected to western blot analysis. As shown in Fig. 6d and e, the phosphorylation levels of mTOR at Ser2448, S6 at Ser235/236, 4E-BP1 at Ser65 and Akt at Ser473 were significantly decreased in tumors from NHPI-treated mice. Taken together, these results illustrate that NHPI inhibits tumor growth in BT-20 xenografts in association with its suppression of both mTORC1 and mTORC2 signaling.

## Discussion

The mTOR signaling pathway is one of the major signaling cascades that regulate cell proliferation and survival. Dysregulation of mTOR signaling pathway is one of the most commonly observed pathological alterations in human cancers [11]. The evidence linking activated mTOR signaling to cancer has generated significant interest in targeting the pathway for cancer therapy [1, 11]. In the present study, we found that NHPI selectively inhibited the proliferation of human breast carcinoma BT-20 cells and human colon adenocarcinoma LoVo cells, concomitantly suppressing mTOR signaling pathway. However, no apparent cytotoxicity was observed in NHPI-treated human normal breast epithelial MCF-10A cells and other tested cancer cell lines, such as human breast adenocarcinoma MDA-MB-231 cells. Interestingly, NHPI at high concentration (20 and 40 μM) did not affect mTOR signaling in MDA-MB-231 cells. Taken together, the above results suggest that the selective proliferation inhibitory effect of NHPI may be attributed to the inhibition of mTOR signaling pathway in these cells.

A plenty of distinct mechanisms including PI3K amplification/mutation, PTEN loss of function, Akt overexpression, and S6K1 or eIF4E overexpression can result in the constitutive activation of the PI3K/Akt/mTOR pathway in cancer cells [7]. We speculate that the distinct mechanisms of mTOR pathway activation in cells seem to be associated with the sensitivity of different cells to NHPI. The fact that the antitumor activity of NHPI is so specific to certain cancer cell lines urged us to focus on the genetic background of these cell lines. Notably, all the three NHPI-sensitive cell lines (BT-20, LoVo and HT-29) harbor mutations of the PI3K p110 catalytic unit, whereas the NHPI-resistant cell lines, such as human breast adenocarcinoma MDA-MB-231 cells, lack mutations of the PI3K p110 catalytic unit. Mutations of the PI3K p110 catalytic unit cause increased PI3K activity that ultimately leads to increased mTOR signaling pathway activity [44, 45]. Thus, we speculate that cell lines with high mTOR signaling pathway activity caused by mutations of the PI3K p110 catalytic unit are more sensitive to NHPI. However, more studies are needed to verify the hypothesis and further elucidate the selective antitumor mechanism of NHPI.

It has been reported that mTOR signaling pathway regulates cell apoptosis and is involved in the up-regulation of survivin via rapid changes in mRNA translation [46, 47]. Our results showed that NHPI induced apoptosis of BT-20 cells by declining the expressions of anti-apoptotic proteins survivin and Bcl-xL as well as activating of caspase 9 and caspase 3. Considering that NHPI inhibited mTOR signaling and decreased expression of anti-apoptotic proteins such as survivin, it is likely that NHPI-induced apoptosis of BT-20 cells is associated with the inhibition of mTOR signaling pathway. Of note, this finding is confirmed in tumors from mice treated with NHPI. In BT-20 xenografts, NHPI significantly inhibited tumor growth. Meanwhile, NHPI reduced mTORC1 activation and its substrate 4E-BP1 phosphorylation as well as the phosphorylation level of Akt at Ser473 in the tumors from NHPI-treated mice, which further suggests that NHPI displays antitumor activity in association with the suppression of both mTORC1 and mTORC2 signaling pathway.

The recognitions that rapalogs have limited substrate-specific efficacy and cause feedback activation of several oncogenic pathways have fostered the development of ATP-competitive mTOR kinase inhibitors (TKIs) [1, 15, 25–27]. The advantage of TKIs is that they inhibit both mTORC1 and mTORC2 [48]. Despite the loss of mTORC2-mediated Ser473 phosphorylation of Akt in cells treated with TKIs, mTORC1 inhibition would still promote feedback activation of PI3K-driven phosphorylation of Akt at Thr308 [3, 15]. Moreover, the loss of mTORC1-mediated IRS-1 feedback might activate PI3K effectors other than Akt [15, 49]. These insights have fueled the development of PI3K and mTOR dual inhibitors. Nevertheless, a recent study showed that NVP-BEZ235, a PI3K and mTOR dual inhibitor, induced the activation of the MAPK as indicated by enhanced ERK phosphorylation, which likely limit the clinical utilization of it in cancer treatment [50–52]. Our present data showed that NHPI effectively inhibited mTORC1 and mTORC2 signaling, and overcame the feedback activation of Akt and ERK caused by mTORC1 inhibition in BT-20 and Lovo cells, indicating that NHPI may achieve a robust anticancer effect.

## Conclusions

In conclusion, we found for the first time that NHPI selectively inhibited the proliferation of human breast carcinoma BT-20 cells, human colon adenocarcinoma LoVo and HT-29 cells, and displayed potent antitumor activity *in vivo* in BT-20 xenografts. The mechanism by which NHPI displays antitumor activity is associated with the inhibition of mTOR signaling pathway. Taken together, our findings suggest that NHPI may be developed as a promising candidate for cancer therapeutics by targeting mTOR signaling pathway.
